# Traditional Widal Agglutination Test Versus Rapid Immunochromatographic Test in the Diagnosis of Enteric Fever: A Prospective Study From South India

**DOI:** 10.7759/cureus.18474

**Published:** 2021-10-04

**Authors:** Praveen R Shahapur, Roopa Shahapur, Anand Nimbal, Tarun Kumar Suvvari, Reewen G D Silva, Venkataramana Kandi

**Affiliations:** 1 Microbiology, BLDE (Deemed to be University) Shri B.M. Patil Medical College, Vijayapur, IND; 2 Dentistry, BLDE (Deemed to be University) Shri B.M. Patil Medical College, Vijayapura, IND; 3 Medicine and Surgery, Dr. N.T.R. University of Health Sciences, Vijayawada, IND; 4 Medicine, Belagavi Institute of Medical Sciences, Belagavi, IND; 5 Clinical Microbiology, Prathima Institute of Medical Sciences, Karimnagar, IND

**Keywords:** diagnosis, immunochromatographic test, widal test, salmonella typhi, typhoid

## Abstract

Introduction

Early diagnosis and treatment are crucial to reducing the morbidity of patients with enteric fever/typhoid fever. Among the available diagnostic tests, the blood culture is considered a gold standard. However, in most of the developing and resource-limited settings, the diagnosis is made utilizing the traditional Widal test and rapid immunochromatographic test (ICT). This study was aimed to compare the diagnostic value and efficacy of ICT and traditional Widal test in the diagnosis of typhoid fever.

Methods

A prospective study was conducted, and 40 patients were included in the study. The Widal test and *Salmonella* *enterica *serovar Typhi IgM/IgG immunochromatographic test were performed for all the patients. The Widal is a tube agglutination test, and the rapid ICT utilizes the principle of enzyme-linked immunosorbent assay (ELISA). All the samples were also tested for the presence of antibodies (IgG and IgM) against the *S*. *enterica *serovar Typhi and the titers against ‘O’ and ‘H’ antigens of *S*. *enterica *serovar Typhi. An antibody titer of 1:80 or more against the ‘O’ and ‘H’ antigen was considered positive.

Results

In the ICT, 24 samples (60%) tested positive for the IgM antibodies, and only 15 samples tested positive and for IgG antibodies. In the Widal test, 27 samples (67.6%) returned positive for antibodies against the *S*. *enterica *serovar Typhi ‘O’ antigen. The sensitivity (90% vs 72.73%), specificity (81.25% vs 64%), and accuracy (82.12% vs 64.87%) for the Widal test were found to be more when compared to the ICT.

Conclusion

The results indicate that the traditional Widal agglutination test is superior to the rapid ICT in the diagnosis of enteric fever. However, both these tests cannot be considered as gold standards for the diagnosis owing to their low positive predictive values.

## Introduction

Enteric fever or typhoid is generally caused by *Salmonella enterica *serovar Typhi and is an important cause of morbidity and mortality in developing countries. Incidences of paratyphoid fevers caused by *S*. *enterica* serovar Paratyphi A, and *S*. *enterica *serovar ParatyphiB with similar clinical presentations to enteric fever have shown an increasing trend in the past decade [1]. The global prevalence of typhoid fever has been estimated to be between 11.9 and 26.9 million cases with reports from across the world including most parts of the African and Asian regions and some parts of Eastern and Southern Europe, and isolated locations of South America and negligible reports from North America [2,3]. The geographic representation of typhoid fever correlates well with its modes of transmission, which includes feco-oral spread by contaminated water and food. Humans act as a reservoir of infection, with reports of chronic carriage and shedding of *Salmonella* in the feces and urine of infected and treated people [4,5]. In 2016, the estimated prevalence of laboratory-confirmed typhoid and paratyphoid fevers across all hospital studies stood at 9.7% and 0.9% respectively, in India [6]. Typhoid is more common among the pediatric age group and affects them the most. This is evident by the community-based cohort studies conducted between 1995 and 2006 in India that estimated the incidence of culture-confirmed typhoid at 377 (178-801) per 100,000 person-years with the highest incidence in early childhood [7].

Clinically, typhoid presents as fever, malaise, abdominal discomfort, diarrhea, constipation, and other nonspecific symptoms, which are often confused with other causes of febrile syndromes [8]. Typhoid can range from mild to severe febrile illness with few other morbidities to marked toxemia and associated multisystem complications. Fever is present in a majority of patients (>90%) irrespective of their age group [9].

The gold standard method to diagnose typhoid fever is by the culture of blood and bone marrow. Urine and feces cultures may also yield growth among patients who became carriers either after clinical infection or after contact with infected persons. Limited facilities, manpower, time, money, and resources hinder diagnosis by culture methods. Therefore, alternative investigations like immunochromatographic tests (ICTs) and Widal tests are used. Other modes of investigations like latex agglutination, co-agglutination, and the polymerase chain reaction (PCR) are also available.

A Widal test is used in endemic areas, but it has low sensitivity and specificity. Widal is an agglutination test that is preferred during the second week of disease to support the clinical diagnosis [10]. Also, due to the low specificity of the Widal test, a fourfold increase in the antibody titer within a gap of one week for the confirmation of typhoid fever is inevitable. Therefore, a single Widal test is less dependable during the diagnosis. Nevertheless, it was recommended to consider a higher antibody titer in a single test for a specific diagnosis of typhoid among infected patients [11].

The ICTs are commercially available and are called by their trade names like Typhidot. ICTs are easy to perform and require no special equipment or training of staff for the interpretation of results and are alternatively used to complement the Widal test and blood culture results [7]. Despite the existing disease burden, the ICT has not been studied well in comparison to the Widal test.

As early diagnosis and treatment are crucial to reducing the morbidity and improving the survival of a patient with typhoid fever, the following study aims to find out the diagnostic value and comparative efficacy of the ITCs in comparison with the traditional Widal test in the diagnosis of typhoid fever among patients who presented to the hospital in the first week of fever.

## Materials and methods

A prospective study was conducted, and 40 patients were included in the study. The informed consent was obtained from all the study subjects and the study was approved by the institutional ethical committee of the BLDE (Deemed to be University) Shri B.M. Patil Medical College. The study included all age group patients who presented with a minimum of two days of fever, abdominal pain/discomfort, diarrhea/constipation, those who were not on antibiotics, and who did not meet the criteria for other common disease diagnoses like malaria, dengue, among others. Patients without fever, gastrointestinal symptoms, those who were on prior antibiotic treatment, and who met the criteria for other common disease diagnoses like malaria, dengue, among others were excluded from the study.

A total of 3 ml of blood was collected from each patient, and after centrifuging, the serum was used to perform the Widal agglutination test (tube method) and *S*. *enterica* serovar Typhi IgM/IgG immunochromatographic test among the patients. Patient’s serum was used to test for the presence of antibodies against *S*. *enterica* serovar Typhi ‘O’ and ‘H’, and *S*. *enterica* serovar​ ​​​​​​Paratyphi ‘AH’ and ‘BH’ antigens using Arkray febrile antigen set (Arkray Inc., Kyoto, Japan) for the Widal tube agglutination test. All the samples were also tested for the presence of antibodies (IgG and IgM) against the *S*. *enterica* serovar Typhi using Enterocheck WB(®). Widal test results were interpreted by demonstrating agglutinating antibody titers against the ‘O’ and ‘H’ antigen of *S*. *enterica* serovar​​​​​​​ Typhi. An antibody titer of 1:80 or more against the ‘O’ and ‘H’ antigen was considered positive.

Statistical analysis

Data were entered into Microsoft Excel 2019, SPSS 24 (IBM Corp., Armonk, NY), and were interpreted for percentages and tables. The calculation of sensitivity, specificity, accuracy, positive predictive value (PPV) and negative predictive value (NPV) was performed using an online resource (https://www.medcalc.org/calc/diagnostic_test.php). The disease prevalence was considered as 11%.

The formulas used for the calculations were as follows:

Sensitivity = True positives/True positives + False negatives

Specificity = True negatives/True negatives + False positives

PPV = Sensitivity × Prevalence/Sensitivity × Prevalence + (1 - Specificity) × (1 - Prevalence)

NPV = Specificity × (1 - Prevalence)/(1 - Sensitivity) × Prevalence + Specificity × (1 - Prevalence)

Accuracy = Sensitivity × Prevalence + Specificity X (1 - Prevalence)

The criteria for the statistical evaluation of the Widal test were as follows:

True positive: T ‘O’ and T ‘H’ positive (n=27)

False positive: Only T ‘H’ positive (n=3)

True negative: T ‘O’ and T ‘H’ negative (n=13)

False negative: Only T ‘H’ positive (n=3)

The criteria for the statistical evaluation of ICT were as follows:

True positive: IgM and IgG positive (n=24)

False positive: Only IgG positive (n=9)

True negative: IgM and IgG negative (n=16)

False negative: Only IgG positive (n=9)

## Results

A total of 40 samples were evaluated; 21 samples (52.5%) belonged to male patients and 19 samples (47.5%) belonged to female patients. The mean age was 26.18 ± 12.94 years and the median days of fever were 6 days (range 2-10 days).

We performed an ICT and 24 samples (60%) tested positive for IgM and 16 samples (40%) tested negative for IgM. For IgG antibodies, only 15 samples (37.5%) tested positive and 25 samples (62.5%) tested negative.

In the Widal test, for ‘O’ titers, 5 samples showed 1:80, 11 samples showed 1:160, 10 samples showed 1:320, and 1 sample showed 1:360. Hence, a total of 27 samples (67.5%) tested positive for antibodies against the *S*. *enterica *serovar ​​​Typhi‘O’ antigen. For ‘H’ titers, 2 samples showed 1:80, 10 samples showed 1:160, and 18 samples showed 1:320. Hence, a total of 30 samples (75%) tested positive for antibodies against the *S*. *enterica* serovar Typhi‘H’ antigen. None of the samples tested positive for *S*. *enterica *serovar Paratyphi A and *S*. *enterica* serovar Paratyphi B.

The sensitivity, specificity, and accuracy of the Widal test in the present study were 90%, 81.25%, and 82.12%, respectively. The sensitivity, specificity, and accuracy of the ICT in the present study were 72.73%, 64%, and 64.87%, respectively, as shown in Table 1.

**Table 1:** Details of the Widal test and immunochromatographic test with reference to their diagnostic efficacy TO: Typhi ‘O’ antibodies; TH: Typhi ‘H’ antibodies; IgG: immunoglobulin G; IgM: immunoglobulin M; PPV: positive predictive value; NPV: negative predictive value *Positive for IgG/IgM with the immunochromatographic test

Typhoid test	TO positive/IgM*	TH positive/IgG*	Sensitivity	Specificity	PPV	NPV	Accuracy
Widal test	27	30	90.00%	81.25%	37.24%	98.50%	82.21%
Immunochromatographic test*	24	15	72.73%	64.00%	19.98%	95.00%	64.96%

## Discussion

Typhoid fever or enteric fever is caused by *S*. *enterica* serovar Typhiand is endemic in the tropical regions of the world. Being transmissible through contaminated food and water, typhoid fever is responsible for frequent infections among both children and adults living in developing and third-world countries. The definitive diagnosis of enteric fever depends on the culture of *S*. *enterica* serovar Typhi from the blood, stool, urine, and cerebrospinal fluid of the infected patients [12]. However, due to the infrastructural, and financial constraints, the diagnosis of typhoid fever is routinely done using the Widal test and rapid ICTs.

The efficacy of both Widal and rapid ICTs has always remained questionable and the debate about the choice of test continues to plague the diagnosis of typhoid fever in the endemic regions. This is mostly due to the patients who present to the hospital late during the infection, and because the patients may be exposed to over-the-counter drugs.

This causes difficulty in the demonstration of fourfold rising titers in the Widal test and false-negative reactions in the rapid ICTs. In the present study that included patients in their first week of infection, the sensitivity, specificity, and accuracy of the Widal test were noted to be superior to the rapid ICTs. However, both revealed low PPVs indicating the possibility of false positives and high NPVs revealing true negative test results [13].

The choice of diagnostic tests is generally made based on the understanding that no positive should be missed, and no negative must be falsely reported [14]. Therefore, both the diagnostic tests compared in the present study are appropriate to be used for the diagnosis of enteric fever. Among them, the traditional Widal test appears to be superior to the rapid ICT based on the high sensitivity, specificity, accuracy, PPV, and NPVs.

The drawback of low PPV includes false-positive diagnosis and unnecessary antibiotic therapy [15]. This could further put a financial burden on the patients and be responsible for the emergence of multi-drug resistance and further limit treatment options [16-21].

In a study by Akter et al., 71 samples were evaluated, and 59.2% of cases were positive for the Widal test and 49.3% of cases were positive for ICT, which was less when compared to our study [22]. The sensitivity and specificity for the Widal test were 100% and 82.9%, respectively, which were a little high when compared to our study. The sensitivity and specificity for the ICT were 88.9% and 91.4%, respectively, which were very high compared to our study [22].

In another study by Danu et al., a total of 100 samples were evaluated and 48% of samples were positive for the Widal test, which is less than the present study [23]. For ICT, 42% of samples reported positive, which is also less than the present study [23]. In a study by Islam et al., the sensitivity and specificity of the Widal test were 83.3% and 80%, respectively, which were less than the present study [24].

In the study by Akter et al., the ICT test showed satisfactory results in terms of sensitivity and specificity compared to the Widal test [22]. But in our study, the sensitivity, specificity, and accuracy of the Widal test were superior to ICT, which corresponded to the findings of previous studies [16,25].

Study limitations

The number of samples included in the study was significantly low, and therefore, the generalization of the results requires further large-scale studies. Also, diagnostic tests were not compared with the gold standard tests like the culture of blood and PCR.

## Conclusions

The present study results demonstrated superior sensitivity, specificity, accuracy, PPVs, and NPVs of the Widal test over the rapid ICTs in the diagnosis of typhoid fever. However, both these tests cannot be considered as gold standards for the diagnosis owing to their low PPVs. Nevertheless, the high NPVs of both the diagnostic tests indicate that the negative test will most certainly rule out infection and avoid needless antimicrobial therapy. The definitive diagnosis of typhoid fever cannot rely solely on serological tests, especially in the endemic regions where the infection is extremely prevalent.
